# The Role of Condensed Tannins in the In Vitro Rumen Fermentation Kinetics in Ruminant Species: Feeding Type Involved?

**DOI:** 10.3390/ani10040635

**Published:** 2020-04-07

**Authors:** Ives C. S. Bueno, Roberta A. Brandi, Gisele M. Fagundes, Gabriela Benetel, James Pierre Muir

**Affiliations:** 1 Department of Animal Science, University of São Paulo, Pirassununga, São Paulo 1365-900, Brazil; ivesbueno@usp.br (I.C.S.B.); robertabrandi@usp.br (R.A.B.); gabriela.benetel@usp.br (G.B.); 2 Department of Animal Science, Federal University of Roraima—UFRR, BR 174, km 12, Boa Vista, Roraima 69300-000, Brazil; 3 Texas A&M AgriLife Research, Texas A&M University, Stephenville, TX 76401, USA; Jim.Muir@ag.tamu.edu

**Keywords:** condensed tannins, fermentability, gas production, grazing ecology, ruminant, microbial responses

## Abstract

**Simple Summary:**

Inoculum from different feeding types of the ruminant species host has unequal tolerance and effects to condensed tannin (CT) due to their respective feeding strategies behavior producing different ruminal microbiota profiles. This paper describes that in long term incubation, CT plant extract addition affects in vitro fermentation kinetics more severely in grazing ruminant than browsing ruminants.

**Abstract:**

Animal feeding behavior and diet composition determine rumen fermentation responses and its microbial characteristics. This study aimed to evaluate the rumen fermentation kinetics of domestic ruminants feeding diets with or without condensed tannins (CT). Holstein dairy cows, Nelore beef cattle, Mediterranean water buffalo, Santa Inês sheep and Saanen goats were used as inoculum donors (three animals of each species). The substrates were maize silage (*Zea mays*), fresh elephant grass (*Pennisetum purpureum*), Tifton-85 hay (*Cynodon* spp.) and fresh alfalfa (*Medicago sativa*). Acacia (*Acacia molissima*) extract was used as the external CT source. The in vitro semi-automated gas production technique was used to assess the fermentation kinetics. The experimental design was completely randomized with five inoculum sources (animal species), four substrates (feeds) and two treatments (with or without extract). The inclusion of CT caused more severe effects in grazing ruminants than selector ruminants.

## 1. Introduction

Tropical shrubs and trees are important feed for livestock because they are sources of protein [1], minerals and vitamins as well as playing important roles in ruminant feeding systems. However, many of these plant species have secondary compounds capable of changing the utilization of nutrients by mammalian herbivores.

Because plants developed defense mechanisms against herbivores and pathogens, animals have developed mechanisms to nullify or restrict the toxic and negative effects of ingested plant secondary compounds such as condensed tannins (CT) [2,3]. Ruminant herbivores and plant CT coexist and adapt natural evolutionary processes. Some ruminant feeders, especially goats, developed physiological adaptations, and even dependence on CT-rich legumes, selectively including such plants in their selector habits [4]. In the case of some browsers such as goats, this adaptation takes the form of salivary glands that produce large amounts of mucus-containing enzymes that can bind to CT to increase palatability and leaving plant proteins more available for digestion [5].

The evolution of different feeding strategies among domestic ruminant species implies differing microbial interactions with CT and, consequently, the diversity of rumen microorganisms and digestive capacity. Thus, we hypothesize that the role of CT on ruminal fermentability may vary depending on the species of the ruminant and, in the case of in vitro fermentation, the species of the rumen fluid donor. Comparative studies with different rumen-fluid donor species with previous pre-adapted to tannin feeding have been published [6,7,8,9]. However, the ability of the rumen microbes of different non-CT-adapted ruminant species to adapt to CT has not been fully investigated. Therefore, the objective of our study was to compare the effects of plant extracts containing CT on ruminal fermentation kinetics of taurine dairy cattle (*Bos taurus taurus*), zebu beef cattle (*Bos taurus indicus*), water buffaloes (*Bubalus bubalis*), sheep (*Ovis aries*) and goats (*Capra hircus*) without previous CT feeding exposure.

## 2. Material and Methods

### 2.1. Animals

Rumen fluid donors included three Holstein dairy cows (*B. taurus taurus*), three Nelore beef cows (*B. taurus indicus*), three Mediterranean water buffalo cows, three Santa Inês ewes and three Saanen goats. Diets were formulated to meet the nutritional requirements of each animal species/breed and consisted of 60% to 80% forage and 20% to 40% concentrate with ground maize and soybean meal. All animals were allowed free access to water and mineral mixture. All methods and animal care were performed in accordance with the relevant guidelines and regulations of the Ethic Committee on Animal Use of the School of Animal Science and Food Engineering, (São Paulo University).

### 2.2. Substrates

Four forages were evaluated as substrates: maize silage (*Zea mays*), fresh elephant grass (*Pennisetum purpureum*), Tifton-85 hay (*Cynodon* spp.) and fresh alfalfa (*Medicago sativa*). Forage samples were dried at 55 °C and ground through a 1-mm sieve. All were analyzed (Table 1) for dry matter (DM), mineral matter (MM), crude protein (CP) and acid-detergent fiber (ADF) with residual ash according to AOAC [10]. Neutral-detergent fiber (NDF) was estimated according to the methodology described by Mertens [11]. Total phenol (TP) concentrations were determined by the Folin-Ciocalteau reagent method [12] and total tannins (TT) were estimated as the difference in TP concentration before and after the treatment with insoluble polyvinylpolypirrolidone [13], using tannic acid as standard. Condensed tannin concentrations were determined by the butanol-HCl method [12], using leucocyanidin as a standard.

Acacia (*Acacia molissima*) extract (Seta^®^ Estância Velha, Brazil) was added to the diets to raise the CT concentration to 50 eq-g of leucocyanidin per kg of feed DM. This CT concentration has been appointed as the minimum to cause harmful effects to ruminants [14]. The chemical characterization of substrates and *Acacia* extract were performed at the Laboratory of Animal Nutrition, Center for Nuclear Energy in Agriculture, University of São Paulo.

### 2.3. In Vitro Gas Production Assay

Ruminal contents were collected through permanent ruminal cannulas from each animal. Equal volumes of liquid and solid phases sample were homogenized in a blender for 10 s. The resulting material was filtered through three layers of cotton (cheese cloth) tissue [15]. Filtered fractions were kept in a water bath at 39 °C and CO_2_ saturation until introduced into the in vitro system.

The in vitro gas production described by Theodorou et al. [16] and Maurício et al. [17] was used to compare disappearance rates. Each 160-mL fermentation flask received 0.5 g of the substrate with or without 0.3 g of extract CT. The inoculation was performed by injecting 10 mL of inoculum in 90 mL of buffered mineral solution [16] into each fermentation flask. The flasks were sealed with rubber stoppers, then shaken and incubated in an oven with forced-air circulation at 39 °C, thus allowing gas accumulation within each flask.

The pressure of the generated gases was measured at 4, 8, 12, 16, 24, 30, 46, 59, 72 and 96 h after inoculation, using a “transducer” (PressData 800^®^). After each measuring, the accumulated gas was released from all bottles. These values were used to calculate the volume of gas produced. At the end of this bioassay (96 h), 2 mL of liquid phase were sampled with a syringe and frozen until analysis of short-chain fatty acids (SCFA). After 96 h, residual material was filtered through sintered crucibles to determine in vitro dry matter degradability (IVDMD) and in vitro organic matter degradability (IVOMD). The partitioning factor (PF), calculated by relating DM degradation and OM degradation to total gas production, was used to compare microbial efficiency [18].

Gas production data were used to determine fermentation kinetics based on the model of Ørskov and McDonald [19] modified by McDonald [20] as:

*p* = 0 to *t* < *t*_0_ (1)

*p* = *a* + *b* (1 − e*xp*^−*ct*^) to *t* ≥ *t*_0_ (2)

where *p* is gas production (ml) in time *t*; *a* and *b* are constants of the model; *c* is the gas production rate (h^−1^); *a + b* is the potential gas production (mL); *t*_0_ is the lag time (h).

### 2.4. Short-Chain Fatty Acids Determination

Short-chain fatty acid analysis was measured by gas chromatography (GC-2014, Shimadzu^®^, Tokyo, Japan), split-injector, flame ionization detector and capillary column (Stabilwax^®^, Restek, Bellefonte, PA, USA) at 145 °C (isothermal) according to Erwin et al. (1961) [21], with adaptations by Getachew et al. [22]. Acetic, propionic, isobutyric, butyric, isovaleric and valeric acid (99.5% purity, Chem service, USA) were used as a quantitative external standard. The operational conditions were: injector and detector temperatures were 250 °C; helium was the carrier gas at 8.01 mL/min; hydrogen flow to the flame jet at 60 kPa and synthetic air at 40 kPa. The samples were thawed at room temperature and centrifuged at 14,500× *g* for 10 min. The supernatant (800 µL) was transferred to a dry and clean flask with 200 µL formic acid (98–100%) and 100 µL of the internal standard (100 mM 2-ethyl butyric acid, Chem service, USA).

### 2.5. Experimental Design and Statistical Analysis

The experiment tested three factors and their interactions. These included four forage substrates, five sources of inoculum (animal species) and two levels of CT extract. Individuals (*n* = 3) of each species constituted the experimental units for three replications.

The statistical design was completely randomized, according to the following model:

Y_ijk_ = μ + F_i_ + S_j_ + T_k_ + (F_i_ × S_j_) + (S_j_ × T_k_) + (F_i_ × T_k_) + e_ijk_ (3)

where Y_ijk_ is the dependent variable; µ the overall mean; F the effect of feeds (substrates) (i = 1 to 4); S the effect of animal species (j = 1 to 5); T the effect of CT (k = 1 to 2); F × S the interaction of feeds and animal species; S × T the interaction of animal species and CT; F×T the interaction of feeds and CT; e_ijk_ the residual error of the model. Results were compared by a Tukey test, using the software SAS for Windows^®^ [23]. Results were considered different at *p* ≤ 0.05.

## 3. Results

### 3.1. Tannin Extract Effects

**Figure 1:**
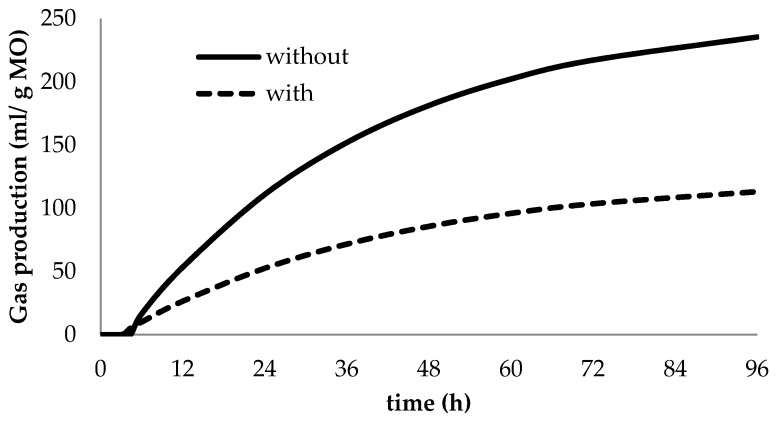
Effects of condensed tannin in diet (with and without) on gas production profiles during in vitro rumen fermentation (pooled over ruminant species).

There was an effect (*p* ≤ 0.001) of CT inclusion on IVDMD and IVOMD regardless of the rumen fluid source. The inclusion of CT inhibited fermentation as indicated by the model parameters (Table 2), visualized in Figure 1. The partitioning factor is a measure created to bring together two variables: degradability and gas production. It was greater (*p* ≤ 0.001) when CTs were included in the diet (Table 2).

Short-chain fatty acids production was also affected by the inclusion of CT extracts (Table 3). The addition of CT promoted a greater (*p* ≤ 0.001) proportion of propionic acid and less (*p* ≤ 0.001) acetic acid.

### 3.2. Animal Species Effects

**Figure 2:**
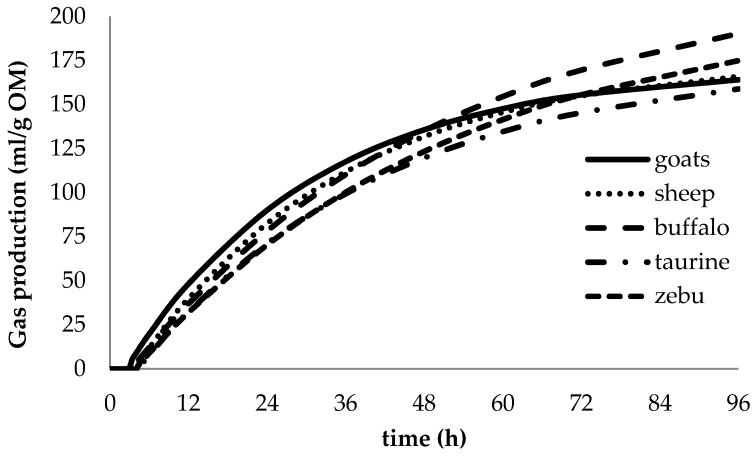
Effects of animal species on gas production profiles during in vitro rumen fermentation (pooled over condensed tannin treatments in four forages).

At the end of the 96-h in vitro fermentation, no differences (*p* > 0.05) in degradability and partitioning factor were observed among animals (Table 4). All species degraded similar proportions of feeds (IVDMD and IVOMD from 0.54 to 0.56) with similar microbial efficiency (PF). However, differences (*p* ≤ 0.01) among model parameters were observed (Figure 2). The production of acetic, propionic and butyric acids by each unit of the degraded substrate was greater (*p* ≤ 0.001) in large than in small ruminants and this was reflected in the total amount of SCFA produced (Table 5). Acetate and butyrate to total acid production were not different.

### 3.3. Interactions

**Figure 3:**
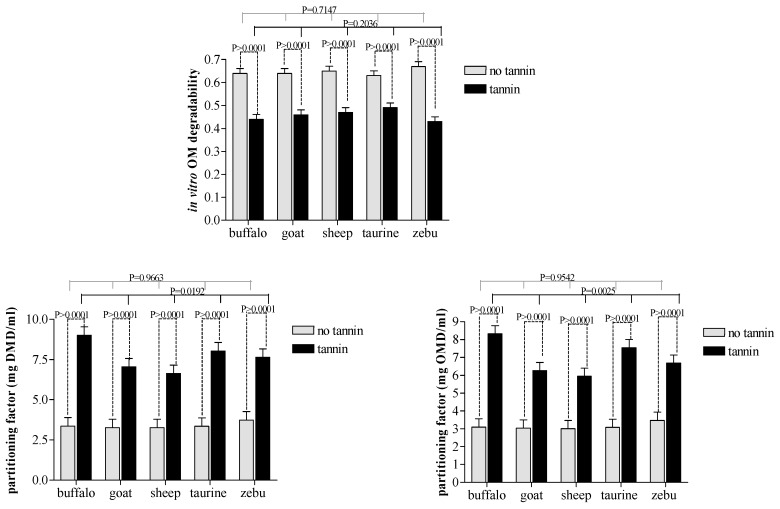
Effects of condensed tannin and animal species interactions on the organic matter degradability and partitioning factor during in vitro rumen fermentation.

CT extract was effective (*p* ≤ 0.001) in reducing IVDMD and IVOMD in all four ruminant species evaluated (Figure 3). However, there were no differences in degradability among animal species between the paired treatment groups receiving CT and those which did not (*p* > 0.05). Among the treatment combinations including CT, buffalo and cattle showed greater microbial efficiency (*p* ≤ 0.01) than sheep or goats (Figure 3). The same was not observed for the partitioning factor by species not receiving CT because all species showed similar values. Condensed tannin addition to the diet increased the partitioning factor in large and small ruminant species.

**Figure 4:**
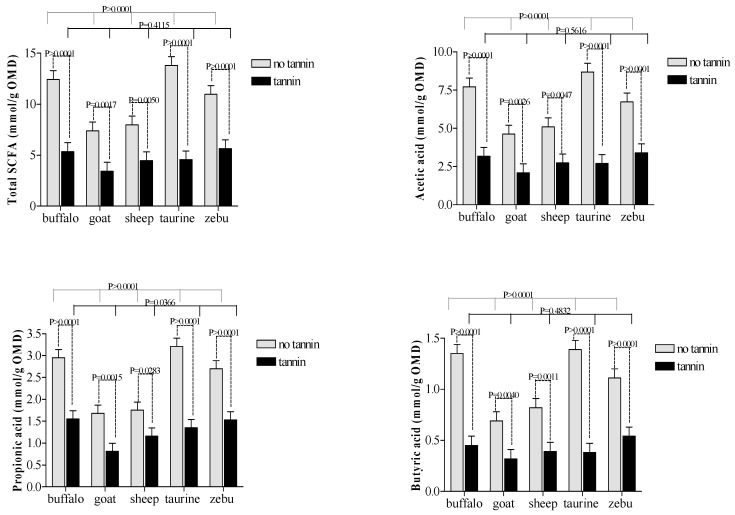
Effects of condensed tannin and animal species interaction on short-chain fatty acids (SCFA) production during in vitro rumen fermentation.

The non-CT diet showed greater (*p* ≤ 0.001) levels of SCFA in cattle and buffalo than in sheep and goats. However, for animals receiving CT, there were no differences (*p* > 0.05) in SCFA between large and small ruminants (Figure 4).

## 4. Discussion

### 4.1. Condensedtannin Extract Effects

Flavonoids from *Acacia* spp. have potential biological effects on rumen fermentation [24]; thus, gas production reduction from diets containing CT extract was expected due to its effect on rumen microbiota. The regression equation for *Acacia* CT extract had a slow initial slope (12 h) followed by a slight increase, which allowed for a constant long-term assay up to 96 h. The slow but steady gas production observed throughout the total incubation period reflects the lower ruminal digestion of organic matter in diets containing CT as reported elsewhere in the literature [25,26]. The inhibitory effects of CT on rumen gas production can be explained by a direct effect of CT-protein complexes on the rumen fibrolytic microbe and consequently lower cell wall degradation. The greater SCFA production and organic matter degradability for tannin-free diets also highlights the interference of CT on cell wall degradability. Previous in vitro studies support our findings that CT may reduce or slow organic matter degradability in the rumen, as reflected in less total SCFA production [27,28,29].

The addition of CT resulted in greater partitioning factors, expressed as mg OM degraded per mL of produced gases. The greater partitioning factor observed in this trial could be due to the initial washing losses by a substrate that might provide greater values of organic matter degradability in poorly substrate degradation samples with CT and therefore with less gas production. However, this assumption needs to be confirmed by future in vitro trials.

### 4.2. Animal Species Effects

Despite their different feeding behavior and digestive capacity, there were similar nutrient degradability and microbial efficiencies among ruminant species studied, which can be explained by the prolonged incubation period of 96 h. The incubation duration used in our study may have allowed the adaptation of the rumen bacterial community to diets, eventually resulting in equivalent substrate fermentation. Contrary to our observations, Calabrò et al. [30] found differences in rumen fermentation between buffaloes and cattle; however, we believe that differences in microbial ecology and their donors probably are strongly reflected in short term incubation [31]. This emphasizes the importance of appropriate incubation duration in studies comparing substrates exposed to inoculants from donors fed different diets.

Due to better fiber digestion capacity, buffaloes, taurine and zebu cattle produced greater amounts of SCFA (per mmol MOD) than small ruminants, regardless of donor diet. Within the ruminant species, goats showed lower production of the major SCFA, including acetic, propionic and butyric acids as well as total SCFA. These results confirm the lower ruminal and omasal capacity and its less efficient degradability of poor quality forages by small-bodied browsers [2,3].

### 4.3. Interactions

The evolution of different feeding behaviors in ruminant species has required anatomic, physiologic and microbial gastrointestinal adaptations to their respective dietary niches [2,32]. In accordance with this hypothesis, Clauss et al. [33] reported clear effects of the ecology of wild ruminants on the dynamics of their protozoal fauna. In this context, the interaction between tanniniferous plants and browsers likely resulted in greater tolerance to flavonoid compounds than in grazers.

The major objective of our study was to assess possible CT-tolerant microbes present in rumen fluid from browsers or grazers. As a result, our 96-h incubation produced different results than those reported for 24 h by Bueno et al. [31], with CT addition resulting in similar total degradation of substrates in vitro during rumen fermentation among grazing and browsing ruminants. In other words, CT was effective in reducing cell wall degradability rates and SCFA production in all species evaluated. This finding suggests that an incubation time of 96 h was insufficient to alter rumen microbial dynamics associated with changing diets, resulting in an incomplete adaptation of ruminal microbial community to the new diet.

Comparisons across CT treatment groups revealed greater propionic acid production and microbial efficiency in buffalo compared to cattle/taurine. Both results suggest that microbial populations in buffalo appear to be less sensitive to dietary CT. Although this contradicts most published research on the topic, it agrees with results reported by Salem [34] that buffalos were more tolerant of CT than other large grazers.

Despite the few differences observed as a result of our longer assay duration, results reported from in vivo animal trials must consider factors other than simply diet. These include anatomical adaptions of the mouth, teeth, salivary glands, body mass and digestive system altering selectivity, forage intake, passage rate and rumination time among the different feeding categories. As a result, degradability trials should account for differentiated rumen fermentation responses in browsers and grazers and selective versus bulk feeders when assaying CT-rich substrates.

## 5. Conclusions

The initial differences in the microbial communities resulting from feeding of donor species provide different responses between large and small ruminants in vitro tannin-rich diet fermentation. Inoculum from sheep and goats is less affected by the addition of CT than buffalo, zebu and taurine.

## Figures and Tables

**Table 1:** Chemical composition of substrates used during the assay of in vitro fermentation kinetics.

Composition	Substrates ^(1)^				
	ALF	ELE	TIF	SIL	ACA
organic matter ^(2)^	916.82	897.75	936.45	964.64	978.86
ether extract ^(2)^	84.04	46.72	57.92	62.98	n.d. ^(5)^
crude protein ^(2)^	278.97	60.28	158.02	82.02	n.d. ^(5)^
neutral-detergent fiber ^(2)^	735.62	770.03	795.29	563.28	n.d. ^(5)^
acid-detergent fiber ^(2)^	510.25	519.52	428.92	332.30	n.d. ^(5)^
acid-detergent lignin ^(2)^	126.69	121.63	133.08	71.35	n.d. ^(5)^
total phenols ^(3)^	13.60	5.47	5.32	10.18	558.63
total tannins ^(3)^	8.14	3.05	2.82	6.58	519.58
condensed tannins ^(4)^	0.25	0.10	0.10	0.15	235.87

^(1)^ ALF: fresh alfalfa; ELE: fresh elephant grass; TIF: Tifton-85 hay; SIL: maize silage; ACA: Acacia tannin extract. ^(2)^ expressed as g/kg DM. ^(3)^ expressed as eq-g tannic acid/kg DM. ^(4)^ expressed as eq-g leucocyanidin/kg DM. ^(5)^ n.d.: not determined.

**Table 2:** Effect of condensed tannins (CT) on degradability, fermentability and microbial efficiency during in vitro rumen fermentation in four ruminant species.

Variables	no CT	CT	SEM ^(1)^	*p*-Value ^(2)^
*in vitro* DM degradability	0.646 ^a^	0.466 ^b^	0.0090	***
*in vitro* OM degradability	0.644 ^a^	0.458 ^b^	0.0095	***
partitioning factor (mg DMD/mL)	3.40 ^b^	7.68 ^a^	0.234	***
partitioning factor (mg OMD/mL)	3.14 ^b^	6.94 ^a^	0.206	***
Model parameters ^(3)^				
a	−27.39 ^b^	−9.06 ^a^	0.816	***
b	281.89 ^a^	133.47 ^b^	7.023	***
c	0.0281	0.0258	0.00130	ns
a + b	254.50 ^a^	124.40 ^b^	7.097	***
t_0_	4.39 ^a^	3.06 ^b^	0.149	***

^(1)^ SEM: standard error of means. ^(2)^ ns: not significant (*p* > 0.05); *: *p* ≤ 0.05; **: *p* ≤ 0.01; ***: *p* ≤ 0.001. ^(3)^ Model of Ørskov and McDonald (1979), modified by McDonald (1981): *p* = *a* + *b* (1 − *exp*^−*ct*^), where *p* = gas production (mL), in time *t*; *a* and *b* = constants of model; *c* = production gas rate (h^−1^); *a* + *b* = potential gas production (mL); *t*_0_ = *lag time* (h) ^a,b^ means followed by distinct superscripts, within rows, are different (Tukey test at *p* ≤ 0.05).

**Table 3:** **Effect** of tannins on short-chain fatty acids (SCFA) production, evaluated during in vitro organic matter rumen fermentation with and without condensed tannins (CT).

Variables	no CT	CT	SEM ^(1)^	*p*-Value ^(2)^
SCFA production (mmol/g OMD)				
acetic acid	6.57 ^a^	2.83 ^b^	0.26	***
propionic acid	2.46 ^a^	1.30 ^b^	0.09	***
iso-butyric acid	0.11 ^a^	0.02 ^b^	0.01	***
butyric acid	1.07 ^a^	0.42 ^b^	0.04	***
iso-valeric acid	0.15 ^a^	0.05 ^b^	0.01	***
valeric acid	0.19 ^a^	0.10 ^b^	0.01	***
total SCFA	10.51 ^a^	4.69 ^b^	0.39	***

^(1)^ SEM: standard error of means. ^(2)^ ns: not significant (*p* > 0.05); *: *p* ≤ 0.05; **: *p* ≤ 0.01; ***: *p* ≤ 0.001. ^a,b^ means followed by distinct superscripts, within rows, are different (Tukey test at *p* ≤ 0.05).

**Table 4:** Effect of animal species on degradability, fermentability and microbial efficiency assayed in four forages during in vitro rumen fermentation.

Variable ^(1)^	Animal Species ^(2)^					SEM ^(3)^	*p* Value ^(4)^
	Goats	Sheep	Buffalo	Taurine Cattle	Zebu Cattle		
IVDMD	0.556	0.565	0.543	0.564	0.554	0.014	Ns
IVOMD	0.551	0.560	0.538	0.559	0.547	0.015	Ns
PF (mg DMD/mL)	5.15	4.94	6.19	5.70	5.69	0.37	Ns
PF (mg OMD/mL)	4.65	4.48	5.70	5.31	5.07	0.33	Ns
Model parameters ^(5)^							
A	−15.15 ^a^	−22.01 ^c^	−15.92 ^ab^	−20.92 ^bc^	−17.13 ^abc^	1.29	***
B	185.82 ^b^	197.70 ^ab^	238.02 ^a^	194.83 ^ab^	222.00 ^ab^	11.10	**
C	0.0348 ^a^	0.0314 ^a^	0.0210 ^b^	0.0267 ^ab^	0.0209 ^b^	0.0021	***
a + b	170.67 ^b^	175.69 ^b^	222.10 ^a^	173.91 ^b^	204.88 ^ab^	11.22	**
t_0_	2.67 ^b^	3.42 ^ab^	4.24 ^a^	4.27 ^a^	4.03 ^a^	0.24	***

^(1)^ IVDMD: in vitro dry matter degradability; IVOMD: in vitro organic matter degradability; GP: gas production; PF: partitioning factor. ^(2)^ breeds—goats: Saanen; sheep: Santa Inês; buffalo: Mediterranea; taurine cattle: Holstein; zebu cattle: Nelore. ^(3)^ SEM: standard error of means. ^(4)^ ns: not significant (*p* > 0.05); *: *p* ≤ 0.05; **: *p* ≤ 0.01; ***: *p* ≤ 0.001. ^(5)^ Model of Ørskov and McDonald (1979), modified by McDonald (1981): *p* = *a* + *b* (1 − *exp*^−*ct*^), where *p* = gas production (mL), in time *t*; *a* and *b* = constants of model; *c* = production gas rate (h^−1^); *a + b* = potential gas production (mL); *t*_0_ = *lag time* (h). ^a,b,c^ means followed by distinct superscripts, within rows, are different (Tukey test at 5%).

**Table 5:** Effect of animal species on short-chain fatty acids (SCFA) production evaluated during in vitro rumen fermentation.

Variables	Animal Species ^(1)^					SEM ^(2)^	*p*-Value ^(3)^
	Goats	Sheep	Buffalo	Taurine Cattle	Zebu Cattle		
SCFA production (mmol/g OMD)							
acetic acid	3.37 ^c^	3.92 ^bc^	5.44 ^ab^	5.70 ^a^	5.06 ^ab^	0.41	***
propionic acid	1.30 ^b^	1.45 ^b^	2.25 ^a^	2.28 ^a^	2.11 ^a^	0.14	***
iso-butyric acid	0.09 ^a^	0.06 ^abc^	0.07 ^ab^	0.02 ^c^	0.04 ^bc^	0.01	***
butyric acid	0.53 ^c^	0.61 ^bc^	0.90 ^a^	0.88 ^a^	0.82 ^ab^	0.07	***
iso-valeric acid	0.10	0.10	0.10	0.12	0.10	0.01	ns
valeric acid	0.12 ^bc^	0.10^c^	0.15 ^abc^	0.19 ^a^	0.17 ^ab^	0.02	***
total SCFA	5.41 ^c^	6.21 ^bc^	8.89 ^a^	9.17 ^a^	8.30 ^ab^	0.61	***

^(1)^ breeds—goats: Saanen; sheep: Santa Inês; buffalo: Mediterranean; taurine cattle: Holstein; zebu cattle: Nelore. ^(2)^ SEM: standard error of means. ^(3)^ ns: not significant (*p* > 0.05); *: *p* ≤ 0.05; **: *p* ≤ 0.01; ***: *p* ≤ 0.001. ^a,b,c^ means followed by distinct superscripts, within rows, are different (Tukey test at *p* ≤ 0.05).
